# Overlapping functions of the MAP4K family kinases Hppy and Msn in Hippo signaling

**DOI:** 10.1038/celldisc.2015.38

**Published:** 2015-11-24

**Authors:** Shuangxi Li, Yong Suk Cho, Tao Yue, Y Tony Ip, Jin Jiang

**Affiliations:** 1 Department of Developmental Biology, University of Texas Southwestern Medical Center, Dallars, TX, USA; 2 Department of Molecular Biology, University of Texas Southwestern Medical Center, Dallars, TX, USA; 3 Center for the genetics and Host Defense, University of Texas Southwestern Medical Center, Dallas, TX, USA; 4 Program in Molecular Medicine, University of Massachusetts Medical School, Worcester, MA, USA; 5 Department of Pharmacology, University of Texas Southwestern Medical Center, Dallars, TX, USA

**Keywords:** Hippo, ISC, MAP4K, organ size, Wts, Yki

## Abstract

The Hippo (Hpo) tumor suppressor pathway is an evolutionarily conserved signaling pathway that controls tissue growth and organ size in species ranging from *Drosophila* to human, and its malfunction has been implicated in many types of human cancer. In this study, we conducted a kinome screen and identified Happyhour (Hppy)/MAP4K3 as a novel player in the Hpo pathway. Our biochemical study showed that Hppy binds and phosphorylates Wts. Our genetic experiments suggest that Hppy acts in parallel and partial redundantly with Misshapen (Msn)/MAP4K4 to regulate Yki nuclear localization and Hpo target gene expression in *Drosophila* wing imaginal discs. Furthermore, we showed that cytoskeleton stress restricts Yki nuclear localization through Hppy and Msn when Hpo activity is compromised, thus providing an explanation for the Wts-dependent but Hpo-independent regulation of Yki in certain contexts. Our study has unraveled an additional layer of complexity in the Hpo signaling pathway and laid down a foundation for exploring how different upstream regulators feed into the core Hpo pathway.

## Introduction

How an organ stops growing when it reaches appropriate size during development is a fascinating but poorly understood problem in modern biology. The control of organ size depends on a delicate balance between cell proliferation and cell death, which are properly coordinated in response to both global and local stimuli. Although tissue growth is influenced by environmental factors such as hormonal signals and nutrients, organ-intrinsic mechanisms also have important roles. The Hippo (Hpo) tumor suppressor pathway has emerged as an evolutionarily conserved signaling pathway that controls tissue growth and organ size in species ranging from *Drosophila* to human, and its malfunction has been implicated in numerous types of human cancer [1–3].

Central to the Hpo pathway is a kinase cascade consisting of an upstream kinase Hpo/MST1/2, members of the Ste20 kinase family [4–8], and a downstream kinase Warts (Wts)/Lats1/2, members of the nuclear Dbf-2-related kinase family [9, 10]. Hpo phosphorylates and activates Wts, which in turn phosphorylates and inactivates Yorkie (Yki), the *Drosophila* homolog of mammalian transcriptional coactivator and oncoprotein YAP/Taz [11]. Phosphorylation of Yki restricts its nuclear localization through recruiting 14-3-3 [12–15]. When the activity of the kinase cascade is compromised, unphosphorylated or under-phosphorylated Yki enters the nucleus and interacts with the TEAD/TEF family transcription factor Scalloped (Sd) to regulate Hpo pathway target genes including *ex*, *cyclin E*, *diap1* and the microRNA *bantam*, which regulate cell growth, proliferation and survival [14, 16, 17].

It is generally thought that the core Hpo pathway is invariant although context dependence of upstream regulators has been documented [18]. However, recent studies revealed that in certain contexts, for example, in response to cytoskeleton stress, Yki/Yap is regulated in a Wts/Lats-dependent but Hpo/MST1/2-independent manner [19, 20], implying that additional kinases may act at the level of Hpo/MST1/2 to regulate Wts/Lats. Indeed, our recent study suggested that Misshapen (Msn)/MAP4K4 regulates Yki/Yap likely through phosphorylating Wts/Lats in *Drosophila* adult midguts [21]. Here we conducted a kinome screen and identified Happyhour (Hppy)/MAP4K3 as a novel player in the Hpo signaling pathway. We provided evidence that Hppy regulates Yki by phosphorylating Wts. We found that Hppy acts in parallel and partial redundantly with Msn/MAP4K4 to regulate Yki nuclear localization and Hpo target gene expression in wing imaginal discs but is dispensable in the regulation of adult *Drosophila* intestinal stem cell (ISC) proliferation. Furthermore, we showed that cytoskeleton stress regulates Yki through Hppy and Msn when Hpo activity is compromised, thus providing a mechanistic explanation for the Wts-dependent and Hpo-independent regulation of Yki in certain contexts.

## Results

### Genetic modifier screen identified Hppy as a new component of the Hpo pathway

**Figure 1:**
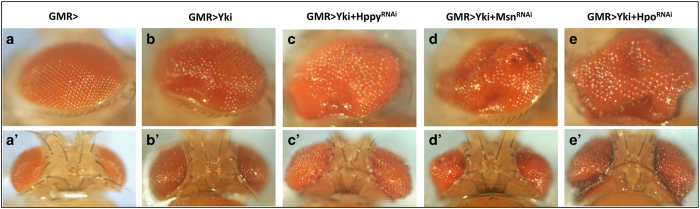
Inactivation of Hppy enhanced the overgrowth phenotype caused by Yki overexpression. (**a**–**e**ʹ) Side (**a**–**e**) and dorsal (**a**ʹ–**e**ʹ) views of adult *Drosophila* eyes of the indicated genotypes. Knockdown of Hppy, Msn or Hpo by RNAi modified the eye overgrowth phenotype induced by Yki overexpression. For Hppy RNAi, the VDRC line, #35166, was used here.

**Figure 2:**
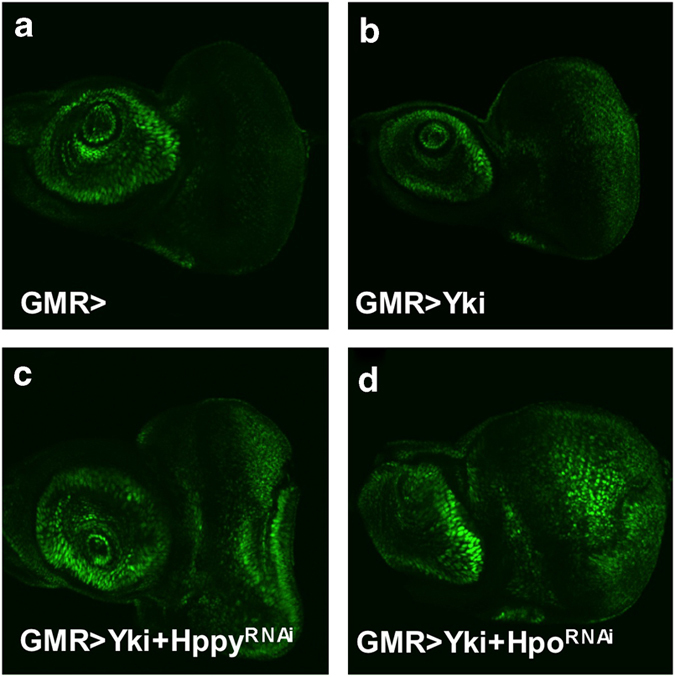
Inactivation of Hppy enhanced Hpo target gene expression driven by Yki overexpression. (**a**–**d**) Eye discs of the indicated genotypes immunostained to show the expression of *diap-GFP*, an Hpo pathway responsive gene. Overexpression of *UAS-Yki* in the posterior region of eye discs by *GMR-Gal4* (*GMR>Yki*) increased *diap-GFP* expression (**b** compared with **a**). Inactivation of Hppy or Hpo by RNAi enhanced *diap-GFP* expression driven by *GMR>Yki*** **(**c** and **d** compared with **a** and **b**).

To identify additional Hpo pathway regulators, we carried out a genetic modifier screen in which we used transgenic RNA interference (RNAi) to inactivate individual genes and determined whether knockdown of the targeted genes modified the overgrowth phenotype caused by Yki overexpression in *Drosophila* eyes (*GMR-Yki*) (Figure 1a and b) [22]. By screening through a collection of transgenic RNAi lines targeting *Drosophila* kinome, we identified several kinases including Hpo, aPKC, Tao1, Msn and Happyhour (Hppy) whose knockdown modified the *GMR-Yki*-induced overgrowth phenotype (Figure 1c–e′; Supplementary Figure S1), although RNAi of Msn or Hppy in otherwise wild-type eye did not affect eye size (Supplementary Figure S2). Because Hppy is the *Drosophila* homolog of mammalian MAP4K3 [23], which is related to Msn/MAP4K4, we went on to explore how Hppy regulates the Hpo pathway. We verified that the loss of Hppy phenotype using two independent RNAi lines, VDRC#35166 and BL#53699, which produced similar results (Figure 1c; Supplementary Figure S1D, data not shown). In addition, we found that Hppy RNAi did not modify the overgrowth phenotype caused by activation of the insulin pathway (*GMR>InR*^*AC*^; Supplementary Figure S3A–C′), suggesting that Hppy regulates tissue growth in an Hpo pathway specificity manner. Furthermore, we found that Hppy RNAi enhanced the expression of *diap-GFP*, an Hpo pathway target gene [14], in eye discs expressing *GMR>Yki* (Figure 2), suggesting that Hppy regulates tissue growth through the Hpo pathway.

### Hppy phosphorylates Wts but not Hpo

**Figure 3:**
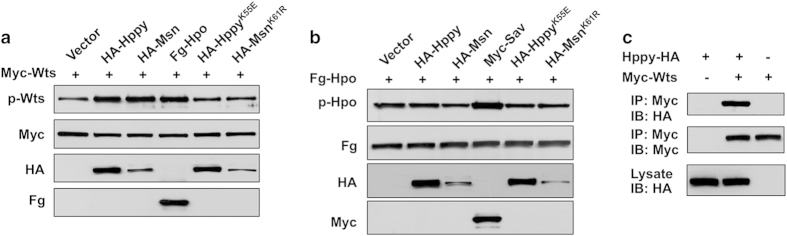
Hppy and Msn promote phosphorylation of Wts but not Hpo. (**a**, **b**) Western blot analysis of cell lysates derived from S2 cells expressing the indicated constructs. Hppy, Msn, as well as Hpo stimulated phosphorylation of Wts at T1077, which was recognized by a phospho-specific antibody p-Wts (**a**). By contrast, neither Hppy nor Msn stimulated phosphorylation of Hpo at T195 recognized by a phospho-specific antibody p-Hpo/MST1/2 (**b**). (**c**) Hppy formed a complex with Wts in S2 cells as revealed by a co-immunoprecipitation assay.

Our previous study showed that, when coexpressed in S2 cells, Msn induced a mobility shift of Wts, which is indicative of Wts phosphorylation [21]. Wts/Lats are phosphorylated and activated by Hpo/Mst1/2 in the hydrophobic loop, which can be detected by a phospho-specific antibody p-Wts (T1077)/p-Lats (T1079) [24, 25]. Using p-Wts, we confirmed that Msn but not its kinase dead form (Msn^K61R^) could promote phosphorylation of the hydrophobic loop of Wts when these proteins were coexpressed in S2 cells (Figure 3a). In line with this observation, our previous study demonstrated that MAP4K4 could also promote phosphorylation of the Lats hydrophobic loop [21].

We went on to determine whether Hppy could also regulate Yki through phosphorylating Wts. Similar to Msn, a wild-type Hppy (HA-Hppy^WT^) but not its kinase dead form (HA-Hppy^K55E^) promoted Wts phosphorylation detected by the p-Wts antibody when Hppy and Wts were cotransfected into S2 cells (Figure 3a). By contrast, neither HA-Msn nor HA-Hppy stimulated Hpo phosphorylation at T195 (Figure 3b). Furthermore, HA-Hppy formed a complex with Myc-Wts detected by a co-immunoprecipitation assay (Figure 3c). Taken together, these biochemical studies suggest that Hppy may act at the level of Hpo similar to Msn.

### Hppy acts redundantly with Msn in the regulation of Hpo signaling in wing discs

**Figure 4:**
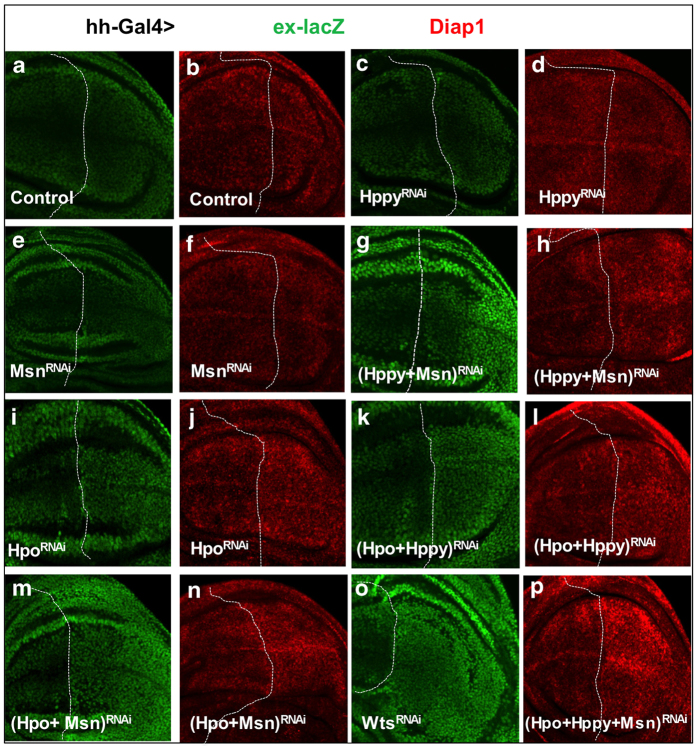
Hppy and Msn act in parallel and partial redundantly with Hpo in wing discs. (**a**–**p**) Late third instar wing discs expressing the indicated RNAi transgenes using *hh-Gal4* were immunostained to show the expression of *ex-lacZ* (green) or *diap1* (red), two Hpo pathway target genes. The discs were orientated with anterior to the left and ventral up. The dashed lines demarcate the anterior/posterior compartment (A/P) boundary determined by co-staining with Ci (Cubitus interruptus) (not shown).

**Figure 5:**
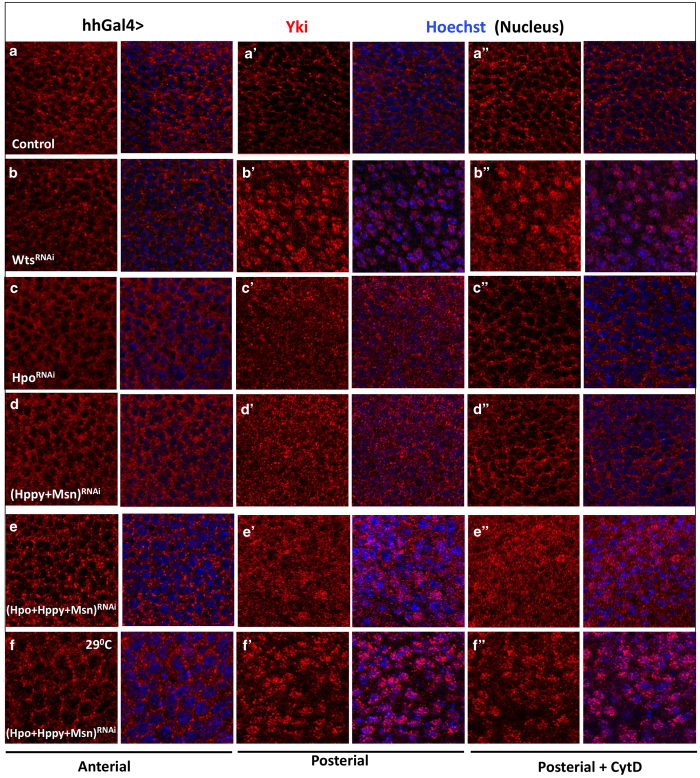
Hppy and Msn mediate Yki nuclear exclusion in response to cytoskeleton stress in wing disc cells with compromised Hpo activity. (**a**–**f**″) High-magnification views of the anterior (A) and posterior (P) compartments of late third instar wing discs expressing the indicated RNAi transgenes with *hh-Gal4* and immunostained with antibodies against Yki (red) and Ci (Cubitus interruptus) (not shown) and a nuclear dye Hoechst (blue). The wing discs were treated with cytochalasin D (+CytD) to disrupt F-actin (**a**″–**f**″). Of note, the wing disc shown in (**f**–**f**″) was derived from larvae grown at 29 °C to increase the expression of RNAi transgenes.

Although Hppy RNAi modified the *GMR>Yki* phenotypes, Hppy knockdown in otherwise wild-type background did not significantly affect Hpo signaling or cell growth in either wing or eye discs (Figure 4c–d compared with Figure 4a and b; Supplementary Figure S2; data not shown). Similarly, Msn knockdown in otherwise wild-type background also did not significantly affect Hpo target gene expression or promote tissue overgrowth (Figure 4e–f; Supplementary Figure S2). It is possible that Hppy and Msn are functionally redundant in the regulation of Hpo signaling in imaginal discs given that they both belong to the MAP4K kinase family. Furthermore, Hpo may have a dominant role in phosphorylating Wts in imaginal discs, which could further mask the function of Hppy and/or Msn in these tissues. To test these possibilities, we examined wing imaginal discs that expressed *UAS-Hppy-RNAi*, *UAS-Msn-RNAi* or *UAS-Hpo-RNAi* in different combinations using the *hh-gal4* driver, which drove the expression of *UAS* transgenes in the posterior compartment of wing discs. Although *hh>Hppy-RNAi* or *hh>Msn-RNAi* did not significantly alter Yki subcellular localization (Supplementary Figure S4C–F′ compared with Supplementary Figure S4A–B′) or the expression of Hpo target genes including *ex-lacZ* and *diap1* (Figure 4c–f compared with Figure 4a and b), combined RNAi of Hppy and Msn increased Yki nuclear localization (Figure 5d and d′ compared with Figure 5a and a′) and elevated expression of *ex-lacZ* and *diap1* in posterior compartments (Figure 4g–h). Furthermore, Hppy or Msn RNAi slightly enhanced the expression of *ex-lacZ* and *diap1* stimulated by Hpo RNAi (Figure 4k–n compared with Figure 4i and j). Finally, simultaneous inactivation of Hpo, Hppy and Msn further increased the expression of *diap1* (Figure 4p) and resulted in a more marked Yki nuclear localization (Figure 5e and e′ compared with Figure 5c and c′, and Figure 5d and d′).

We noticed that Yki nuclear localization in Hpo, Hppy and Msn triple knockdown discs was still less profound compared with that in Wts RNAi discs (Figure 5b and b′). One likely explanation is that RNAi did not completely inactivate Hpo, Hppy and/or Msn so that the residual kinase activity could still phosphorylate Wts to restrict Yki nuclear localization. To test this possibility, we decided to carry out RNAi experiments at 29 °C, which increased the level of transgene expression driven by the *Gal4/UAS* system. Indeed, simultaneous expression of *UAS-Hpo-RNAi*, *UAS-Hppy-RNAi* and *UAS-Msn-RNAi* by *hh-Gal4* at 29 °C resulted in Yki nuclear localization in P-compartment cells similar to those caused by Wts RNAi (Figure 5f and f′ compared with Figure 5b and b′). Moreover, Hpo, Hppy and Msn triple knockdown discs grown at 29 °C exhibited an overgrowth phenotype similar to that of Wts RNAi discs (Supplementary Figure S5; Figure 4o). Taken together, these results suggest that Hppy and Msn act partial redundantly and in parallel with Hpo to regulate Yki activity.

### Hppy is not required in enteroblasts or enterocytes for the regulation of ISC proliferation

We have previously shown that Msn and Hpo have distinct roles in *Drosophila* adult midgut homeostasis in that Msn mainly acts in enteroblasts (EBs), whereas Hpo primarily acts in enterocytes (ECs) to restrict the Yki activity and ISC proliferation [21]. Indeed, inaction of Msn in EBs or inaction of Hpo in ECs by RNAi resulted in increased ISC proliferation (Supplementary Figure S6). By contrast, we found that inactivation of Hppy in either EBs or ECs did not affect ISC proliferation (Supplementary Figure S6), suggesting that Hppy may not have a significant role in the regulation of Hpo signaling in the context of midgut homeostasis.

### Hppy and Msn mediate the Hpo-independent regulation of Yki in response to cytoskeleton stress

Previous studies revealed that cytoskeleton stress regulated Yki/Yap through Wts/Lats1/2 but not Hpo/MST1/2 [19, 20]. Consistent with this notion, we found that treating wing discs with cytochalasin D (CytD), which disrupted F-actin polymerization (Supplementary Figure S4P–Q′) [19], relocated Yki from nuclei to the cytoplasm in Hpo knockdown but not in Wts knockdown wing disc cells (Figure 5b″ and c″ and Supplementary Figure S5I and I′ compared with Figure 5b′ and c′ and Supplementary Figure S5H and H′). As Hppy and Msn act in parallel with Hpo in wing discs, it is possible that Hppy and/or Msn may mediate the effect of CytD on Yki subcellular localization in cells with compromised Hpo activity. Indeed, we found that combined inactivation of Hpo, Hppy and Msn blocked CytD-mediated nuclear exclusion of Yki (Figure 5e″ and f″ compared with Figure 5e′ and f′), whereas CytD treatment could still relocate Yki from the nuclei to the cytoplasm in Hppy+Msn, Hpo+Hppy or Hpo+Msn double RNAi wing discs (Figure 5d″ compared with Figure 5d′; Supplementary Figure S4L and L′ compared with Supplementary Figure S4K and K′), suggesting that these three kinases act partial redundantly to mediate the effect of cytoskeleton stress on Yki activity.

## Discussion

**Figure 6:**
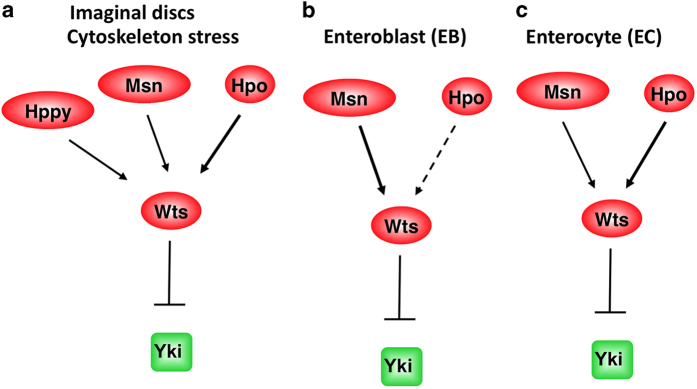
Distinct but overlapping roles of Hppy, Msn and Hpo in the regulation of Wts/Yki. (**a**) In imaginal discs, Hpo has a dominant role, whereas Hppy and Msn have minor roles in the regulation of Wts and Yki; however, in response to cytoskeleton stress, these kinases act in parallel to activate Wts and inhibit Yki. (**b**) In the enteroblasts of *Drosophila* adult midguts, Msn has a dominant role, whereas Hpo has little if any role in restricting Yki activity. (**c**) In enterocytes, Hpo has a major role, whereas Msn a minor role in inhibiting Yki.

In this study, we identified Hppy as a new component in the Hpo signaling pathway. We provided genetic evidence that Hppy and Msn act partial redundantly to regulate Yki nuclear localization and activity in wing discs and that these two MAP4K family kinases act in parallel with Hpo to regulate Yki activity, as well as the growth of imaginal tissues. Our biochemical experiments revealed that Hppy can form a complex with Wts and promote the phosphorylation of the hydrophobic loop of Wts similar to Msn, and that neither kinase promoted the phosphorylation of Hpo activation loop, providing evidence that these MAP4K family kinases regulate Yki activity at the level of Hpo and upstream of Wts (Figure 6). Hence, our study unraveled an additional layer of complexity in the Hpo signaling pathway and laid down a foundation for exploring how different upstream Hpo pathway regulators feed into the core pathway.

Our findings that Hppy and Msn can act in parallel with Hpo to regulate Wts/Yki provide an explanation why in certain contexts the regulation of Yki/Yap depends on Wts/Lats but not Hpo/Mst1/2. For example, knockout of Mst1/2 did not affect Yap phosphorylation in MEF cells [26, 27], and cytoskeleton stress could stimulate Lats/Yap phosphorylation and exclude Yap from the nuclei in Mst1/2 mutant cells [20]. These observations suggest that an additional kinase(s) may act in parallel with Hpo/Mst1/2 to promote Wts/Lats phosphorylation. Indeed, our previous study revealed that MAP4K4 knockout blocked cytoskeleton stress-induced Lats/Yap phosphorylation [21]. Here we showed that simultaneous inaction of both Msn/MAP4K4 and Hppy/MAP4K3 could block cytoskeleton stress-induced nuclear exclusion of Yki in Hpo knockdown cells (Figure 5), suggesting that these MAP4K family kinases act redundantly with Hpo to restrict Yki nuclear localization in response to cytoskeleton stress.

Our study also provided new insight into the context-dependent regulation of Hpo signaling (Figure 6). Our findings suggest that Hpo has a major role in imaginal disc development, as well as in ECs of adult midguts, whereas Msn has a dominant role in EBs to restrict Yki activity. Although Hppy has a minor role in imaginal disc development and acts partial redundantly with Msn and Hpo to mediate the effect of cytoskeleton stress on Yki nuclear localization, its activity is not required either in ECs or EBs for the regulation of ISC proliferation. It would be interesting to explore in the future how tissue- or context-dependent regulation is achieved. There are a large number of MAP4K family members in mammals. Our study raises the possibility that different MAP4K family members may be employed to regulate Hpo signaling in different developmental and homeostatic contexts.

While this manuscript was in preparation, two independent studies reported that Hppy/Msn/MAK4K family kinases act in parallel and partial redundantly with Hpo/Mst1/2 to regulate Wts/Lats1/2 [28, 29]. In agreement with our findings, Meng *et al.* [29] found that MAP4K4 could phosphorylate the hydrophobic loop of Lats and that loss of Msn function by RNAi slightly enhanced the expression of *ex-lacZ* in *hpo* mutant clones in wing discs. In addition, these authors showed that combined knockout of MAP4K4, MAP4K6, MAP4K7, MST1 and MST2 (MM-5KO) diminished Latrunculin B (LatB)-stimulated Yap phosphorylation, suggesting that the mammalian homologs of Msn act redundantly with MST1/2 to mediate the effect of cytoskeleton stress on Lats/Yap phosphorylation. By contrast, Zheng *et al*. [28] showed that Msn did not promote Wts phosphorylation. The reason for this discrepancy remains unclear. Zheng *et al.* [28] also showed that LatB induced nuclear exclusion of Yki in *hpo* mutant clones but *hpo hppy* double-mutant clones were largely unresponsive to LatB in wing discs, suggesting that Hppy acts redundantly with Hpo to mediate the effect of cytoskeleton stress on Yki . However, these authors also noticed that LatB could induce nuclear exclusion of Yki in 15% of the *hpo hppy* double-mutant clones [28]. The incomplete penetrance of the *hpo hppy* double-mutant phenotype is likely due to the activity of Msn. Indeed, we found that combined inactivation of Hpo, Hppy and Msn by RNAi resulted in more robust Yki nuclear localization than any double inactivation, and that only triple but not any double knockdown conferred resistance to CytD-induced Yki nuclear exclusion (Figure 5; Supplementary Figure S4). We propose that a certain threshold of kinase activity contributed by Hpo, Hppy and Msn is required to mediate the effect of cytoskeleton stress on Wts/Yki phosphorylation and activity.

Hpo is activated by its binding partner Sav [30], and Sav is further regulated by the cell adhesion molecule Echinoid [22]. However, the upstream activators of Hppy and Msn remain unclear. It would be interesting to determine whether cytoskeleton stress could regulate the activity of Hppy and Msn as our previous study demonstrates that actin regulating proteins can affect intestinal homeostasis via Msn [21]. Another important question that needs to be addressed in the future is why some tissues/cell types rely on one kinase more than the others, for example, EBs rely on Msn more than Hpo, whereas wing disc cells rely on Hpo more than Msn and Hppy. The finding that overexpression of Hpo in EBs failed to rescue loss of Msn suggested that Hpo might not be activated effectively in these cells [21], although the underlying mechanism remains to be determined in the future.

## Materials and Methods

### Drosophila genetics and transgenes

Flies were raised on standard yeast/molasses medium at 25 °C. Transgenic RNAi lines used for this study: *UAS-Hppy-RNAi* (VDRC#35166 and BL#53699); *UAS-Msn-RNAi* (BL# 28791 and VDRC#101517); *UAS-Hpo-RNAi* (BL#33614 and VDRC#104169); *Wts RNAi* (VDRC#106174); *UAS-aPKC-RNAi* (BL#25946), *UAS-Tao1-RNAi* (VDRC#34881). Transgenes: *UAS-Yki* [14]; *UAS-InR*^*AC*^ (BL#8263); *UAS-HA-Msn* and *UAS-HA-Msn*^*K61R*^ [21]; *UAS-HA-Hppy* and *UAS-HA-Hppy*^*K55E*^ [31], *UAS-Myc-Wts* and *UAS-Flag-Hpo* [8]. Gal4 driver: *GMR-Gal4* (flybase); *hh-Gal4* [14]; *Su(H)*^*t*s^ and *Myo1A*^*ts*^ [21].

### Immunostaining

Immunostaining of imaginal discs was carried out using standard protocol as previously described. For cytochalasin D treatment, wing discs were dissected out from late third instar larvae in 1× phosphate-buffered saline (PBS), incubated in S2 medium containing 20 µg ml^−1^ cytochalasin D (Sigma, St Louis, MO, USA) for 1 h, and then fixed with 4% paraformaldehyde for 20 min before immunostaining. Female flies were used for gut immunostaining in all experiments. The entire gastrointestinal tract was taken and fixed in 1× PBS plus 8% EM grade formaldehyde (Polysciences, Warrington, PA, USA) for 2 h. Samples were washed and incubated with primary and secondary antibodies in a solution containing 1× PBS, 0.5% bovine serum albumin and 0.1% Triton X-100. To generate Yki antibody, full-length Yki-coding sequence was cloned into the *pET* vector. His-Yki protein was expressed in BL21(DE3) *Escherichia coli* (Invitrogen, Carlsbad, CA, USA) by induction with 1 mm isopropyl β-d-1-thiogalactopyranoside. Insoluble inclusion bodies containing the majority of His-Yki protein were used to immunize rabbits (Cocalico Biologicals, Reamstown, PA, USA). The specificity of rabbit anti-Yki antibody was confirmed by western blot and immunostaining. Other primary antibodies used in this study were: rabbit anti-βGal (MP Biomedicals, Santa Ana, CA, USA), 1:1 000; rabbit anti-Diap1 [32], 1:100; rabbit and mouse anti-PH3 (Millipore, Billerica, MA, USA), 1:1 000; goat anti-GFP (Abcam, Cambridge, UK), 1:5 000, Hoechst (Life Technologies, Carlsbad, CA, USA), 1:500. Alexa Fluor-conjugated secondary antibodies were used at 1:400 (Jackson ImmunoResearch and Invitrogen, West Grove, PA, USA). Discs and guts were mounted in 70% glycerol, and imaged by a Zeiss confocal microscope (Zeiss LSM 710 inverted confocal, Zeiss, Oberkochen, Germany) using ×20 or ×40 oil objectives (imaging medium: Zeiss Immersol 518F, Zeiss). The acquisition and processing software were Zeiss LSM Image Browser, and image processing was performed in Adobe Photoshop CC.

### Cell culture, transfection, immunoprecipitation and western blot analysis

*Drosophila* S2 cells were cultured in *Drosophila* SFM (Invitrogen) with 10% fetal bovine serum, 100 U ml^−1^ of penicillin and 100 mg ml^−1^ of streptomycin at 24 °C. Transfections were carried out using the Calcium Phosphate Transfection Kit (Specialty Media). Immunoprecipitation and western blot analysis were carried out as previously described [33]. Antibodies used were as follows: mouse anti-Myc (Santa Cruz Biotechnology, Santa Cruz, CA, USA, sc-40); mouse anti-HA (Santa Cruz Biotechnology, sc-7392); mouse anti-Flag (Sigma, M2 F3165); rabbit anti-p-Hpo/Mst1/2 (Cell Signaling Technology, Danvers, MA, USA, #3681). The phospho-specific antibody against Wts-T1077 was a gift from Dr DJ Pan [34].
