# Supplementary material for: Overlapping functions of the MAP4K family kinases Hppy and Msn in Hippo signaling
Source: Cell Discov. 2015 Nov 24;1:15038–. doi: 10.1038/celldisc.2015.38 (PMC4860773; doi:10.1038/celldisc.2015.38)
Supplement: Supplementary Figures [file celldisc201538-s1.pdf]

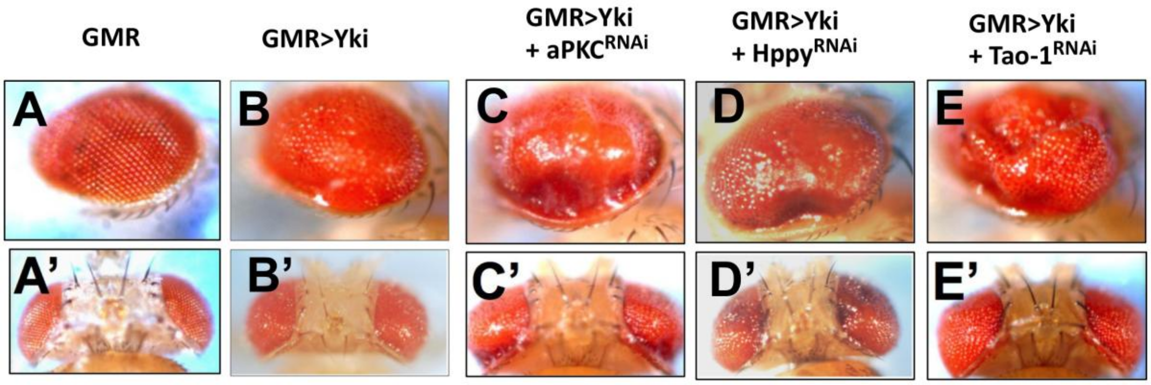


**Figure S1 Genetic modifier screen to identify new components in Hpo signaling**

(A-E') Side (A-E) and dorsal (A'-E') views of adult *Drosophila* eyes of the indicated genotypes. Overexpression of *UAS-Yki* using an eye specific gal4 driver *GMR-Gal4* (*GMR>Yki*) resulted in enlarged eyes (compare B-B' with A-A'). Flies carrying *GMR>Yki* were crossed to *UAS* transgenic RNAi lines targeting individual kinases and their progenies were screened for modification of the eye phenotype. Representatives of genetic enhancers were shown in C-E'. For Hppy RNAi, the Bloomington line, BL#53699, was used here.

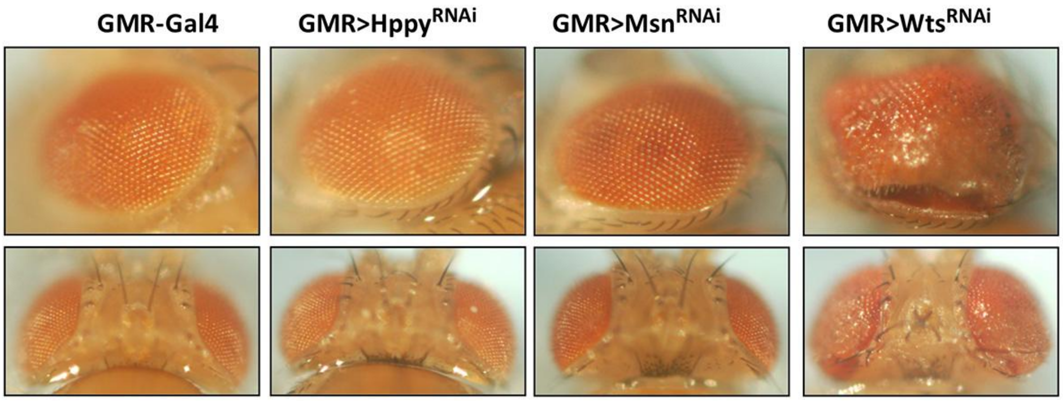


**Figure S2 RNAi of Hppy or Msn using *GMR-Gal4* did not alter eye size**

Side (top) and dorsal (bottom) views of adult *Drosophila* eyes of the indicated genotypes. Unlike knockdown of Wts, which caused eye overgrowth, knockdown of Hppy or Msn using *GMR-Gal4* did not alter eye size.

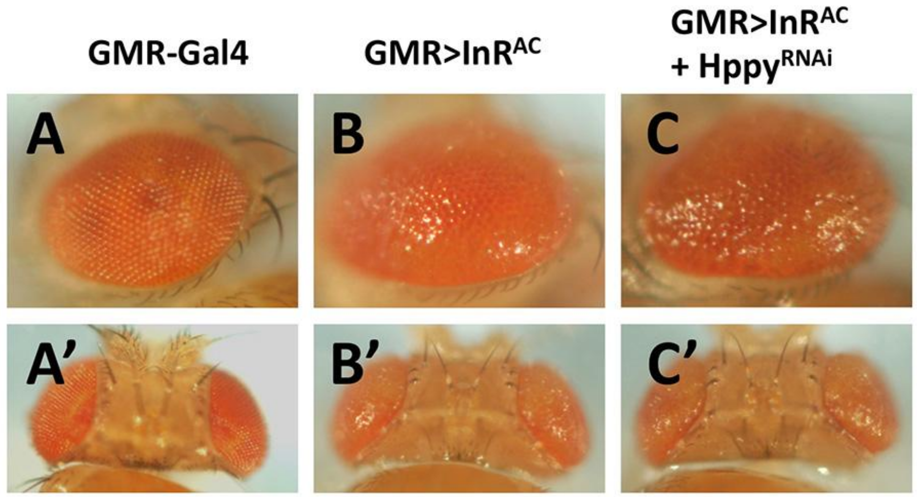


**Figure S3 Inactivation of Hppy did not enhance the eye phenotype caused by activation of insulin pathway**

(A-C') Side (A-C) and dorsal (A'-C') views of control eyes (A, A') or eyes expressing a constitutively active form of insulin receptor ($InR^{AC}$; B-B') or $InR^{AC}$ together with $Hppy^{RNAi}$ with *GMR-Gal4* (C-C'). Hppy RNAi did not significantly enhance the eye phenotype caused by constitutively activating the insulin pathway.

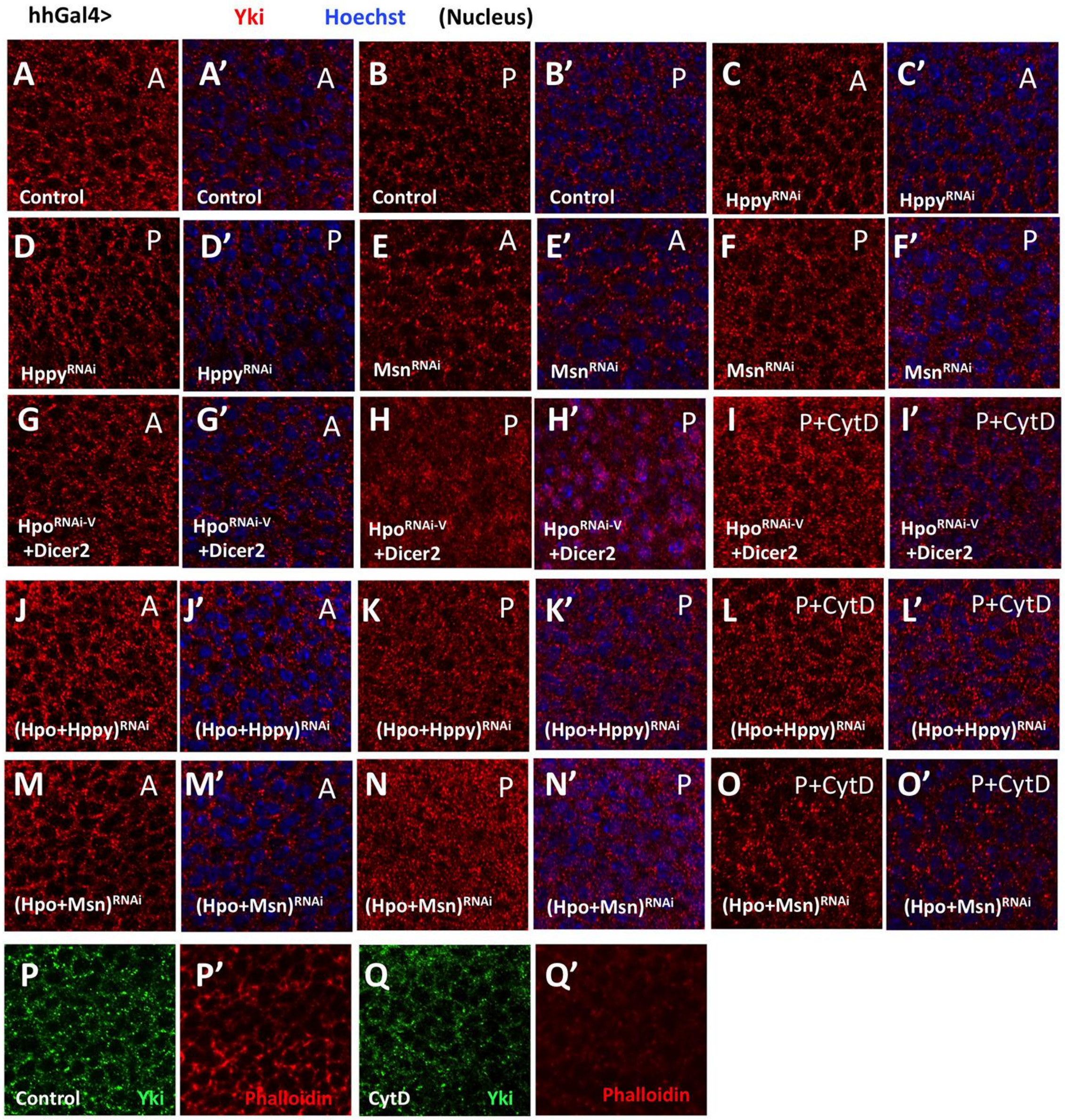


**Figure S4 Hppy and Msn act redundantly with Hpo to regulate Yki nuclear localization in response to cytoskeleton stress**

(A-Q) High magnification views of anterior (A) and posterior (P) compartments of late third instar wing discs expressing the indicated RNAi transgenes with the *hh-Gal4* driver and immunostained with antibodies against Yki (red) and Ci (not shown) and a nuclear

dye Hoechst (blue). To determine the effect of cytoskeleton stress on Yki nuclear localization, the wing discs were treated with cytochalasin D (CytD) to disrupt F-actin. Of note, $Hpo^{RNAi-V}$ is a VDRC line that produces long dsRNA whose RNAi effect can be enhanced by coexpression of Dicer 2, whereas $Hpo^{RNAi}$ is a Bloomington line that produces shRNA. (P-Q') Control or Cyt-D treated wing discs were stained for Yki (green) and phalloidin (red), which marks F-actin. Cyt-D treatment disrupted F-actin polymerization.

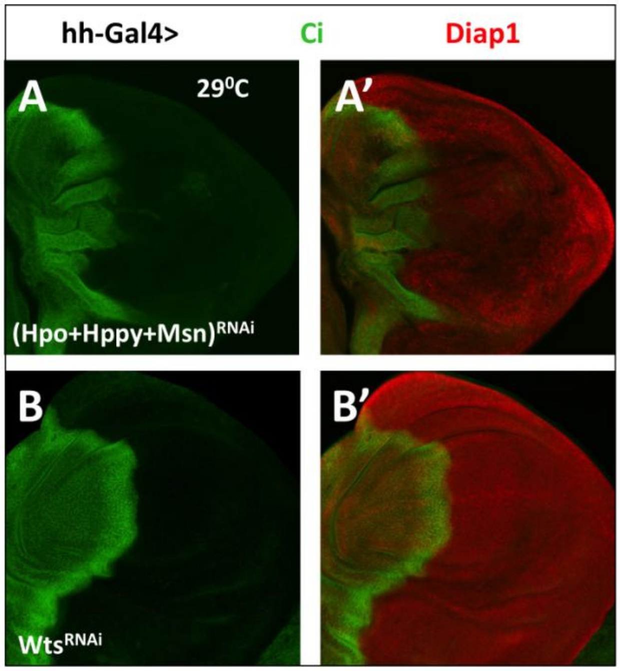


**Figure S5 Simultaneous inactivation of Hpo, Hppy and Msn resulted in tissue overgrowth similar to inaction of Wts**

(A-B') Late third instar wing discs expressing Hpo, Hppy, and Msn RNAi transgenes with the *hh-Gal4* driver at $29^0$C (A-A') or Wts RNAi (B-B') were immunostained with antibodies against Diap1 (red) and Ci (Green). Ci staining marks the anterior compartment of wing discs. Inactivation of Wts or simultaneous inactivation of Hpo, Hppy and Msn in posterior compartment cells resulted in a dramatic overgrowth of the posterior compartments.

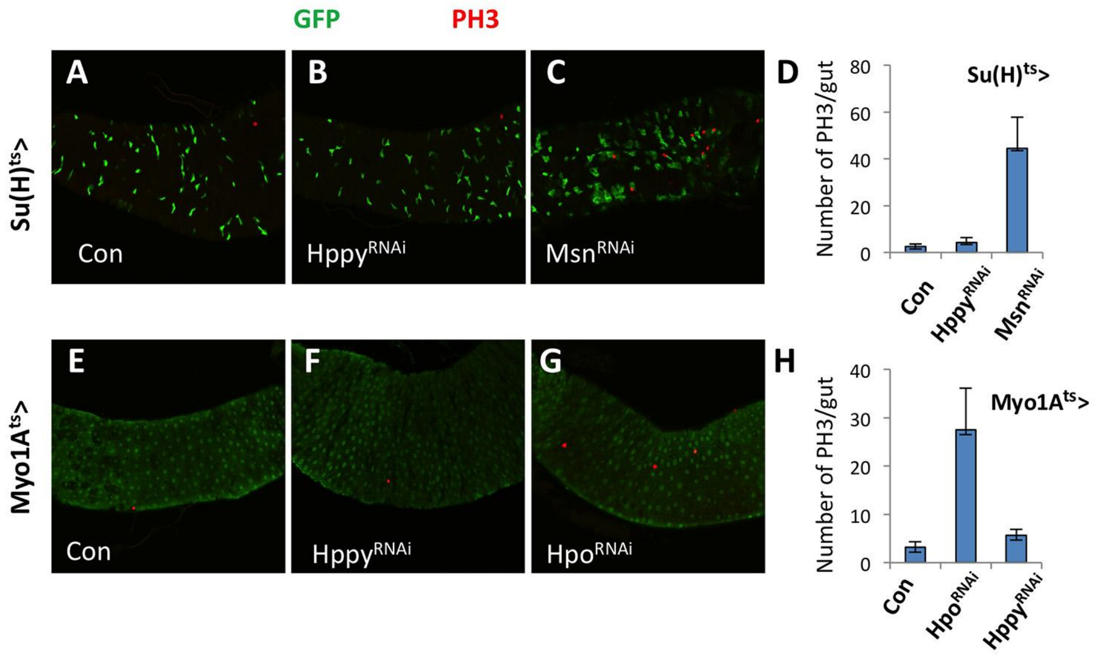


**Figure S6 Inactivation of Hppy in enteroblasts or enterocytes does not affect intestinal stem cell proliferation**

(A-C, E-G) PH3 staining in control adult midguts (A, E), midguts expressing *UAS-Hppy-RNAi* or *UAS-Msn-RNAi* using the enteroblast-specific gal4 driver *Su(H)*$^{ts}$ (B, C), or *Hppy-RNAi* or *UAS-Hpo-RNAi* using the enterocyte-specific Gal4 driver *Myo1A*$^{ts}$ (F, G) at 30$^{o}$C for 7 days. (D, H) Quantification of PH3$^{+}$ cells in midguts of the indicated genotypes treated. Two independent experiments were performed and 20 guts were examined for each sample per experiment. Error bars are standard deviations.
